# Arachidonic Acid‐Induced Contraction of Smooth Muscle Is Mediated by MLC Phosphorylation in Preterm Birth

**DOI:** 10.1155/jp/8266780

**Published:** 2026-03-18

**Authors:** Xinyi Chen, Jing Chen, Jin Qiu, Yan Yan, Runjie Zhang

**Affiliations:** ^1^ Obstetrics and Gynecology Department, Tongren Hospital, Shanghai Jiao Tong University School of Medicine, Shanghai, China, shsmu.edu.cn; ^2^ Hongqiao International Institute of Medicine, Shanghai Jiao Tong University School of Medicine, Shanghai, China, shsmu.edu.cn

**Keywords:** arachidonic acid (AA), cervical smooth muscle contraction, phosphorylated MLC, preterm birth, RhoA/ROCK signaling pathway

## Abstract

Preterm birth (PTB) remains a challenging issue in the reproductive field, and cervical maturation is an essential physiological prerequisite for parturition. The cervix is rich in smooth muscle cells, and their abnormal contractility is a key trigger for premature cervical remodeling, which may further lead to spontaneous PTB. Herein, we found that high serum arachidonic acid (AA) expression in PTB mice may predict potential harms through untargeted metabolomics analysis. After AA intervention, immunofluorescence/qPCR/WB revealed that the expression of cervical smooth muscle contraction indexes calponin/oxytocin receptor (OR)/connexin‐34 and premature birth‐related factors cyclooxygenase‐2 (COX‐2) increased significantly, indicating that AA acting on cervical smooth muscle cells may lead to premature birth. WB results showed that the expression of phosphorylated myosin light chain (p‐MLC) in cervical smooth muscle cells treated with AA increased significantly, and myosin light chain (MLC) protein was closely related to smooth muscle contraction. After adding the Ras homolog gene family member A (RhoA)/Rho‐associated coiled‐coil forming protein kinase (ROCK) pathway inhibitor, the expression of p‐MLC decreased significantly, indicating that AA could induce MLC phosphorylation through the RhoA/ROCK signaling pathway to cause cervical smooth muscle shrinkage and lead to premature birth. In summary, our findings provided evidence that AA enhanced cervical smooth muscle contraction and led to PTB by inducing MLC phosphorylation through the RhoA/ROCK signaling pathway. Hence, our study provided new insights into mechanisms linking cervical smooth muscle contraction to PTB muscle shrinkage, suggesting that AA could be a potential novel drug intervention target for PTB therapy.

## 1. Introduction

Preterm birth (PTB) is a complex syndrome induced by multiple high‐risk factors that remains the leading cause of morbidity and mortality in perinatal infants and children under 5 years [1]. Moreover, premature infants are at increased risk of neurological disorders and other chronic diseases in adulthood, which has a significant financial and social impact on families and communities [2].

A growing body of evidence suggested that the development of preterm labor involves multiple biological pathways, including intrauterine infection, fetal stress, extracellular matrix (ECM) degradation, and estrogen metabolic pathways. Currently, the use of uterine contraction‐inhibiting drugs is the main clinical treatment for PTB, which delays delivery but still does not reduce the incidence of PTB. Premature cervical remodeling, softening, and dilation are common pathways to eventual preterm vaginal birth [3]. miR‐143 and miR‐145 expression in the interstitial cervical space disrupted the cervical epithelial barrier and played a major role in cervical remodeling in PTB [4]. It has been found that the internal os of the cervix contains a large amount of contractile smooth muscle similar to the “sphincter” that can respond to contractile agonists such as adrenergic receptor agonists and oxytocin, independent of uterine contractions [5], and may lead to premature cervical shortening and increase the risk of PTB. Another study pointed out the presence of longitudinally and circularly arranged muscle fibers in the cervix, with more contractions of longitudinal muscle fibers located medially in the cervix contributing to cervical dilation and contractions of circular muscle fibers located laterally in the cervix hindering cervical dilation and keeping the cervix closed [6]. Meanwhile, the hardness of the ECM affected the contractility of human cervical smooth muscle cells (CSMCs), and soft ECM may reduce CSMC contractility and predispose to soft cervical dilation, which led to the occurrence of PTB [7]. In addition, it was found that inflammation may lead to smooth muscle remodeling, which in turn contributed to proliferation and fibrosis, and increased the secretion of smooth muscle contraction signals (oxytocin), resulting in uterine smooth muscle contraction [8]. Mechanisms of the inflammation effect on cervical smooth muscle leading to cervical remodeling causing PTB remain to be clarified and may vary among different reasons needing further studies.

Arachidonic acid (AA; n‐6, 20:4) is a long‐chain polyunsaturated *ω*‐6 fatty acid, one of the most abundant essential fatty acids in the human body. It can be obtained directly from the diet or synthesized by linoleic acid through a series of actions. AA can be metabolized by three different enzyme systems, including cyclooxygenase (COX), cytochrome P450 (CYP) enzyme, and lipoxygenase (LOX), producing a variety of biologically active fatty acids [9]. Among them, the COX enzyme was the earliest reported to metabolize AA, which was divided into two subtypes: COX‐1 was mainly involved in physiological functions such as platelet aggregation, and COX‐2 primarily took part in pathophysiological processes such as inflammation and cancer [10]. AA produced prostaglandins (PGs), including PGs and thromboxane A2 (TXA2), through the action of the COX enzyme, which stimulated uterine contractions to cause or miscarriage [11]. Studies have found that free AA in amniotic fluid increased during miscarriage and delivery, resulting in an increased synthesis of PGF2a, PGE2‐prostacyclin, and TXA2 in fetal membranes and villi [12]. Nevertheless, the mechanism of action of AA in CSMC and PTB is not clear.

The purpose of this study was to ascertain the effect of AA on CSMC and to illuminate the mechanisms by which AA moderates human CSMC function. Within this study, we authenticated the influences of AA on CSMC shrinkage to investigate how AA regulates CSMC shrinkage by inducing MLC (myosin light chain) phosphorylation through the RhoA/ROCK signaling pathway, leading to the onset of PTB.

## 2. Materials and Methods

### 2.1. Animal Experimental Protocols and Sample Preparation

In each cage, 10–11‐week‐old C57BL/6 mice were housed at a female to male ratio of 2:1. The mice were maintained under a strictly regulated 12‐h light/dark cycle. The environmental humidity was fixed at 50%–60%, and the temperature was maintained at 21^°^C ± 2^°^C. Pregnancy was detected by visually inspecting for the presence of a vaginal plug. The day on which the vaginal plug was identified was designated as gestational Day 0 (Day 0). Subsequently, pregnant mice were randomly allocated into two experimental groups: the control group received an intraperitoneal injection of phosphate‐buffered saline (PBS), whereas the PTB group received an intraperitoneal injection of lipopolysaccharide (LPS, manufactured by Biotech Co. Ltd., United States) on gestational Day 15.5. After the initial injection of either PBS or LPS, the mice were monitored hourly for signs of labor, including reduced activity, vaginal bleeding, and preterm delivery. The onset of preterm delivery was defined as the birth of the first pup. Most mice in the PTB group started giving birth 10–18 h postinjection, with the majority delivering between 10 and 12 h. Once labor commenced in the PTB group, all mice were humanely euthanized. Blood samples and cervical tissues were then collected. All collected samples were from mice at the same gestational age. A total of 1‐mL venous blood per mouse was drawn from the tail vein. The blood was centrifuged at 3500 rpm for 10 min at 4°C. The supernatant was then transferred to a 1.5‐mL centrifuge tube and stored as a serum sample for LC–MS detection. Each frozen serum sample was thawed separately at room temperature. After labeling the cervical tissues, the specimens were cut into serial macroscopic slices, which were used for immunohistochemical staining.

All the mice were sourced from the experimental animal center of the Minhang campus of East China Normal University. This animal study was comprehensively reviewed and approved by the Ethics Committee of Animal Experiments at Shanghai Tongren Hospital, Shanghai Jiao Tong University School of Medicine.

### 2.2. Cell Culture

Human CSMCs (#EH0089) were purchased from Shanghai Yu Chun Biotechnology Company. CSMCs were cultured in DMEM/F‐12 (Gibco, United States) culture medium containing 10% fetal bovine serum (FBS, Gibco, United States) and 1% PSA (penicillin G, streptomycin, amphotericin B, Gibco, United States), in a humid environment of 37°C and 5% CO2. The culture medium was changed per 2–3 days, limiting the culture to five generations.

### 2.3. Purchase of AA

AA (#T4129‐506‐32‐1) was purchased from TargetMol (United States). The 100‐mM AA stock solutions were prepared in dimethyl sulfoxide (DMSO). These AA stock solutions were then diluted to 20 *μ*M using the culture medium, keeping the DMSO concentration below 0.1%, and added to the intervention group for 48 h. Next, an equal amount of DMSO was added to the control group.

### 2.4. Staining

#### 2.4.1. Immunohistochemical staining

After dehydration with graded ethanol and transfer to xylene, the cervical tissues were embedded in paraffin. The sections were heated in a citrate‐phosphate buffer (pH 6.0) for antigen retrieval, followed by treatment with 3% hydrogen peroxide to block endogenous peroxidase activity. Next, the sections were blocked with 10% normal goat serum at room temperature for 30 min to prevent nonspecific binding. The sections were then incubated with primary antibodies (connexin‐43, oxytocin receptor (OR), cyclooxygenase 2 (COX‐2), and calponin) diluted in PBS at 4°C for 24 h. After several washes with PBS, the sections were incubated with secondary antibodies at room temperature for 30 min. For the negative control, PBS was used in place of the primary antibody. A total of nine pregnant dams were included in the study, with three biological replicates per experimental group (three groups in total). Each sample was subjected to three technical replicates.

#### 2.4.2. Immunofluorescence staining

Cells were evenly plated in a 24‐well plate and seeded on glass slides. After treatment with metabolites, the sections were fixed, rinsed with PBS, and incubated in a blocking solution containing Triton×100 and bovine serum albumin in PBS for 2 h at room temperature. The sections were then incubated with primary antibodies overnight at 4°C. The sections were washed with PBS and incubated with a secondary antibody for 1 h at room temperature in the dark. Finally, the sections were rinsed again with PBS, mounted using fluoroshield mounting medium containing DAPI (Cat. No. P0131, Beyotime, China), and imaged by a fluorescence microscope. The cell culture experiments consisted of three independent biological sets (*n* = 3 per group, three groups total), corresponding to three separate cell seeding and treatment batches.

The information of the primary antibodies was as follows: connexin‐43 polyclonal antibody (Cat No. 26980‐1‐AP, Proteintech, United States), OR polyclonal antibody (Cat No. 23045‐1‐AP, Proteintech, United States), COX‐2 monoclonal antibody (Cat No. 66351‐1‐Ig, Proteintech, United States), and calponin monoclonal antibody (Cat No. 13938‐1‐AP, Proteintech, United States).

The information of the secondary antibodies was as follows: CoraLite488‐conjugated Goat Anti‐Mouse IgG(H+L) (Cat No. SA00013‐1, Proteintech, United States) and CoraLite488‐conjugated Goat Anti‐Rabbit IgG(H+L) (Cat No. SA00013‐2, Proteintech, United States).

### 2.5. Detection of Metabolic Profiling by LC–MS

Untargeted metabolomics was analyzed by LC–MS. The main system comprised an Ultimate 3000 UHPLC (Thermo Fisher, United States) coupled to a Thermo Orbitrap Elite MS, with ESI source (positive/negative ion mode) and mass resolution of 70,000 at m/z 200. MS/MS used data‐dependent dd‐MS2 (Top *N* = 10), full‐scan resolution 17,500 at m/z 200, and scan range 100–1500 m/z. ESI+ and ESI− metabolic profiles were acquired via ACQUITY UPLC I‐Class (Waters, United States) linked to AB SCIEX Triple TOF 5600 MS (AB SCIEX, United States). Binary gradient elution: mobile Phases A (0.1% formic acid in water, v/v) and B (0.1% formic acid in acetonitrile, v/v). Elution program: initial 20% B sustained for 2 min, followed by ramping to 60% B over 4 min, then increasing to 100% B with an 11‐min hold. After maintaining 100% B until 13 min, the mobile phase was switched to 5% B at 13.5 min and held until the 14.5‐min mark. Chromatographic conditions: injection volume 2 *μ*L, column temp. 25°C, flow rate 0.35 mL/min. Data were collected in centroid mode with Thermo Excalibur 2.2 software (Thermo Fisher, United States).

### 2.6. Quantitative Polymerase Chain Reaction (qPCR)

Cell total RNA was extracted with TRIzol reagent (Invitrogen, United States), and total RNA was reverse‐transcribed into complementary DNA strands (cDNA) using a Reverse Transcription Kit (Takara, Japan). The target genes were replicated using SYBR Green PCR Master Mix (Applied Biosystems, United States). The PCR primers are depicted in Table 1. The amplification conditions were as follows: 50°C for 2 min, 95°C for 2 min, 40 cycles at 95°C for 15 s, and 60°C for 60 s. Normalize the quantified mRNA values to GAPDH as a control. The 2‐*ΔΔ*Ct method was used to determine the relative expression of genes. The cell samples used consist of three independent biological sets (*n* = 3 per group, three groups total), corresponding to three separate cell seeding and treatment batches.

**Table 1:** List of primers.

Gene	The gene accession number	Sequence	Sequence (5 ^′^ → 3 ^′^)
Calponin	NM_001299.4	F	AGGTTAAGAACAAGCTGGCCC
		R	ATGAAGTTGTTGCCGATGCG
COX‐2	NM_000963.4	F	TCCCTTGGGTGTCAAAGGTAAA
		R	TGGCCCTCGCTTATGATCTG
Oxytocin receptor (OR)	NM_000916.4	F	GACTCGGTGCAGTGGAAGC
		R	CTGAGCCACTGCAAATGAGC
Connexin‐43	NM_000165.4	F	CTGAGTGCCTGAACTTGCCT
		R	CCTGGGCACCACTCTTTTGC

*Note:* F represents forward primer; R represents reverse primer.

### 2.7. Western Blotting

Cells were lysed in 100 *μ*L of cold RIPA buffer containing protease inhibitors (Thermo Fisher Scientific, United States). The cell samples used consist of three independent biological sets (*n* = 3 per group, three groups total), corresponding to three separate cell seeding and treatment batches. The total protein content was quantified with the BCA Protein Concentration Determination Kit (Beyotime, China). Samples were dissolved in 5× protein loading buffer (Epizyme, China), dry boiled at 95°C for 10 min, and electrophoresed on 4%–12% SDS‐PAGE gels (Smart‐Lifesciences, China). The protein was transferred to the nitrocellulose membrane and blocked with 5% skim milk powder after electrophoresis. Blots were probed at 4°C overnight with anti–*α*‐SMA (1:1000, 14395‐1‐AP, Proteintech, United Kingdom), anti‐SM22 (1:1000, 10493‐1‐AP, Proteintech, United Kingdom), anti–COX‐2 (1:1000, 12375‐1‐AP, Proteintech, United Kingdom), anti–connexin‐43 (1:1000, 26980‐1‐AP, Proteintech, United Kingdom), anti‐calponin (1:1000, 13938‐1‐AP, Proteintech, United Kingdom), and anti‐OR (1:1000, 23045‐1‐AP, Proteintech, United Kingdom). Blots were incubated for 1 h at RT with HRP‐conjugated Goat Anti‐Rabbit IgG (H+L) (1:5000, SA00001‐2, Proteintech, United Kingdom) after three washes with TBST. GAPDH (1:10000, HRP‐60004, Proteintech, United Kingdom) was used as a standard control protein. Density analysis using computer imaging software (ImageJ) quantified western blotting results.

### 2.8. Molecular Docking

We obtained the 3D structure of AA (CAS No.: 506‐32‐1, HMDB id: HNBD0001043) from the PubChem website (https://pubchem.ncbi.nlm.nih.gov). The minimum RMS gradient set in SDF format was 0.001, and the optimized structure was saved in mol2 format. Next, through the stitch database (http://stitch.embl.de/), the protein RhoA, which interacts with AA, was identified. The optimized small molecule was imported into AutodockTools‐1.5.6 for hydrogenation, charge calculation, charge distribution, and setting of rotatable bonds, and then saved in “pdbqt” format. Subsequently, the protein RhoA (PDB ID: 6V6U) was downloaded from the RCSB‐PDB database (https://www.rcsb.org), and protein crystal water, original ligands, and other nonessential components were removed using PyMOL 2.3.0 (DeLano Scientific LLC, United States). The protein structure was then imported into AutoDockTools 1.5.6 (Scripps Research Institute, United States) for hydrogenation, charge calculation, charge distribution, atom type assignment, and saved in “pdbqt” format. Next, the protein binding site was predicted by POCASA 1.1 (Google Inc., United States), and molecular docking was performed by AutoDockVina 1.1.2 (Scripps Research Institute, United States). The parameters for RhoA were set as follows: center_x = 3.7, center_y = 3.5, and center_z = 2.2; search space dimensions: size_x = 60, size_y = 60, and size_z = 60 (with a grid spacing of 0.375 Å); exhaustiveness = 10, and other parameters were set to default. Finally, the interaction mode of the docking results was analyzed using PyMOL 2.3.0. Additionally, the top‐ranked docking conformation (selected based on binding energy) was subjected to interaction analysis using LigPlot+ software.

### 2.9. Statistical Analysis

All results were expressed as mean ± standard deviation (SE). All data analysis was processed using GraphPad Prism Version 6.0 (GraphPad Software, San Diego, California) for one‐way ANOVA of differences between the AA and NC groups. The comparison between the two groups used the unpaired Student′s *t*‐test. *p* value < 0.05 was considered statistically significant. ∗*p* < 0.05, ∗∗*p* < 0.01, ∗∗∗*p* < 0.001, and ∗∗∗∗*p* < 0.0001.

### 2.10. Confirmation of Compliance With Wiley′s Artificial Intelligence–Generated Content (AIGC) Policy

We confirm that the submitted manuscript fully adheres to Wiley′s Best Practices Guidelines on Research Integrity and Publishing Ethics regarding the use of AIGC tools based on large language models (LLMs).

## 3. Results

### 3.1. Characteristics and Changes of PTB Mice

To explore the potential role of endogenous metabolites in PTB, inflammation‐induced PTB models were conducted, and compared with the normal group, the fetuses of the premature group were translucent and not fully developed (Figure 1a), indicating models were successfully constructed. Once premature delivery was observed, blood samples were collected and metabolomics analysis. We performed immunohistochemical staining, and immunohistochemistry staining of cervical slices displayed the content of COX‐2, OR, calponin, and connexin‐43 were significantly increased in the PTB group (Figure 1b,c).

Figure 1Phenotypic and histological characteristics of PTB mice. (a) The image shows that the appearance of the mice is immature in the PTB group. (b) Immunohistochemical staining was performed on mouse cervical tissue. The slides show that the cervical tissue of PTB mice contained a large amount of connexin‐43, OR, COX‐2, and calponin. (c) Immunohistochemistry semiquantitative analysis showed that the levels of connexin‐43, OR, COX‐2, and calponin in the PTB group were higher than those in the NC group. (∗∗*p* < 0.01 and ∗∗∗*p* < 0.001).

**Figure:**
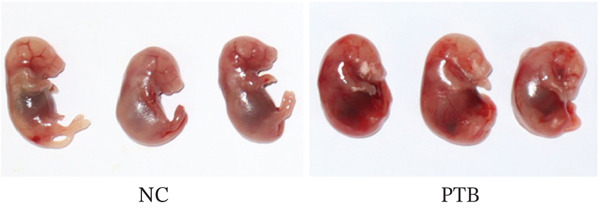
(a)

**Figure:**
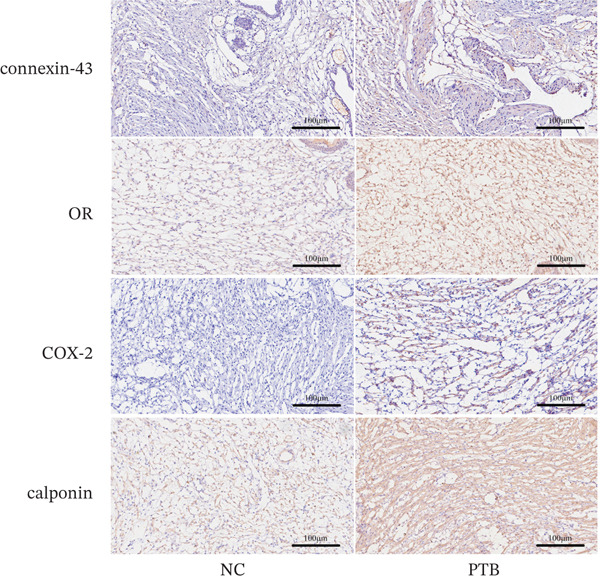
(b)

**Figure:**
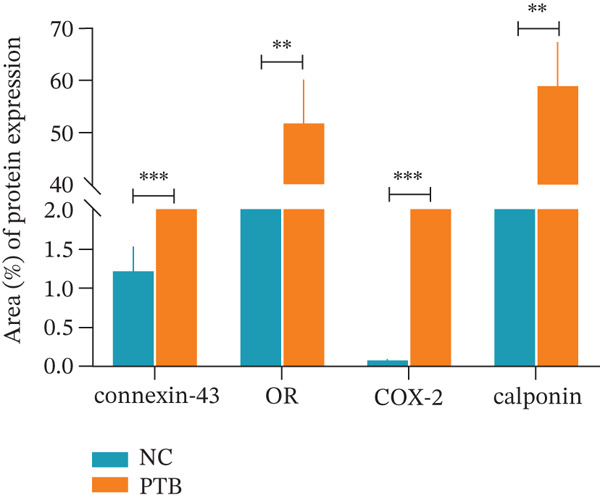
(c)

### 3.2. Identification and Screening of Differentially Expressed Metabolites

In our previous study [13], a total of 181 differential metabolites were identified in the PTB group, of which 96 metabolites were significantly upregulated, and 85 metabolites were significantly downregulated. Based on the metabolomics profile and data about inflammation‐induced PTB and some relevant metabolites in regulating premature cervical ripening in our previous study, we screened out a series of differentially expressed metabolites (Table 2) according to the conditions of *p* < 0.05, VIP (variable importance in the projection) > 1.03 and fold change > 0.60.

**Table 2:** Metabolites in the plasma of PTB mice (*p* < 0.05).

Name	VIP	*p*	Fold change
Arachidonic acid	1.37592	0.00140203	2.40873948
PE(17:0/20:0)	1.45132	0.00098465	3.42288838
PS(20:0/0:0)	1.22808	0.00170475	2.04651660
PC(18:1(9Z)/0:0)	1.08323	0.02489807	1.86003547
3‐Hydroxybutyric acid	1.20368	0.00164398	5.43128927
Acetyl‐L‐carnitine	1.54582	0.00040390	1.95857706
Linoleic acid	1.51085	0.00000664	2.21958847
Dihomo prostaglandin	1.17448	0.00233723	1.45828915
L‐phenylalanine	1.23973	0.00658132	1.40514864
Taurine	1.04184	0.02623258	0.72756711
D‐(‐)‐glutamine	1.03624	0.02901402	0.69064811
L‐Lysine	1.62264	0.00008174	0.60103405

Then, we conducted a literature review about these candidate metabolites. Referring to Katayama′s article that focused on the effects of AA on contraction of smooth muscle via myosin motor domain [14], Serra′s research found AA could activate the RhoA/ROCK pathway within human coronary artery smooth muscle cells [15] and Masataka′s opinion that biologically active lipids such as AA are known to affect smooth muscle contractility [16], AA with increased content in the PTB mice was selected as candidate metabolites to explore its potential biological functions.

### 3.3. Potential Biological Functions of Metabolites in Human CSMC

To delve deeper into the biological significance of metabolites, we identified candidate metabolites and treated human CSMC with AA. The effects of AA were evaluated with immunofluorescence staining. AA enhanced the expression of connexin‐43, OR, COX‐2, and calponin in the human CSMC (Figures 2a, 2b, 2c, and 2d). After treatment with AA, the expression of mRNA and protein of connexin‐43, OR, COX‐2, and calponin in human CSMC increased (Figures 3a, 3b, and 3c). All the changes were statistically significant.

Figure 2The effects of AA on human CSMC were evaluated with immunofluorescence staining. (a–d) AA enhanced the expression of connexin‐43, OR, COX‐2, and calponin in the human CSMC. The cell nucleus was stained with DAPI.

**Figure:**
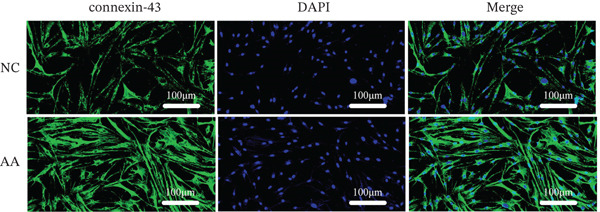
(a)

**Figure:**
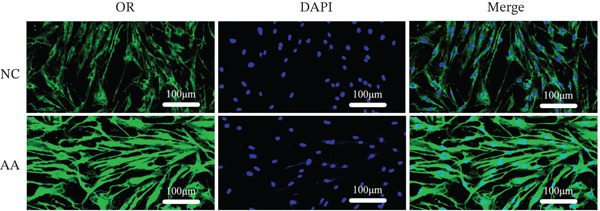
(b)

**Figure:**
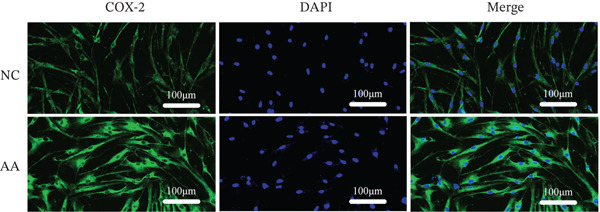
(c)

**Figure:**
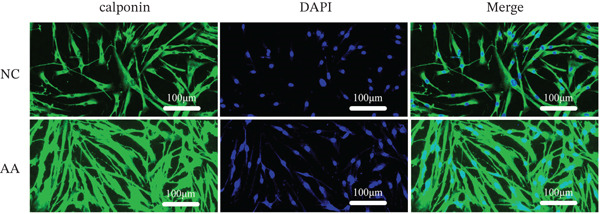
(d)

Figure 3The impact of AA on human CSMC. (a) The mRNA levels of connexin‐43, OR, COX‐2, and calponin in human CSMC increased after treatment with AA. (∗∗∗*p* < 0.001 and ∗∗∗∗*p* < 0.0001). (b) Western blotting analysis of connexin‐43, OR, COX‐2, and calponin protein levels in human CSMC after treatment with AA. (c) Densitometric analysis of western blots. (∗*p* < 0.05).

**Figure:**
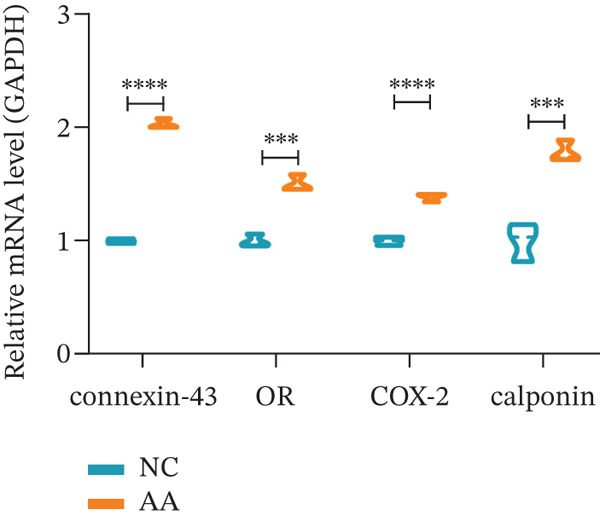
(a)

**Figure:**
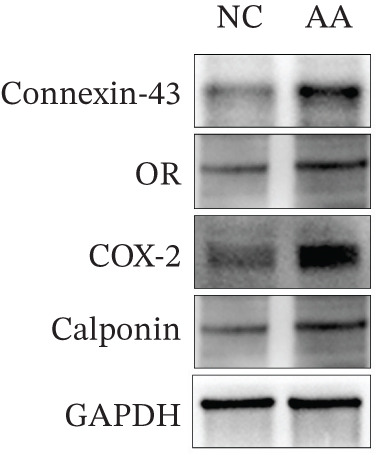
(b)

**Figure:**
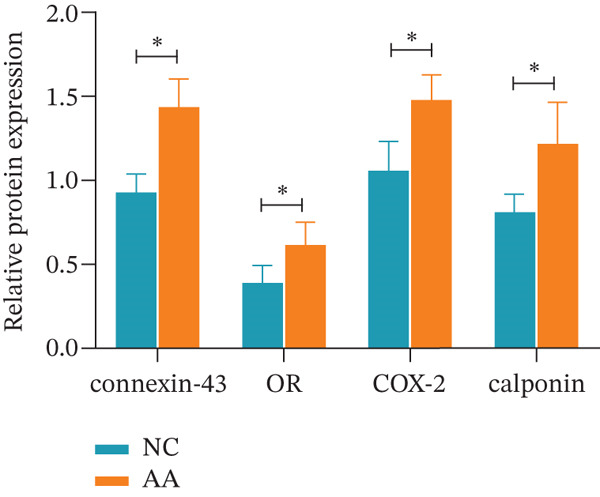
(c)

### 3.4. Mechanism of AA in Human CSMC

Molecular docking experiments indicated that the binding energy of AA and RhoA is −6.1 kcal/mol, which proves that it has a good binding effect. AA forms a hydrogen bond with LEU (leucine)‐57 of RhoA with a length of 2.8 Å (Figures 4a, 4b, and 4c). LigPlot+ analysis revealed the presence of key intermolecular interactions; except for hydrogen bonds, multiple hydrophobic interactions were observed between the ligand and hydrophobic residues of the protein, including Leu72(A), Ala61(A), Tyr66(A), Ala56(A), Trp58(A), and Leu69(A), which contribute to the stability of the ligand–protein complex. Additionally, Van der Waals interactions were observed between the ligand and residues of the protein, involving Asn41(A), Asp59(A), Thr60(A), Ser73(A), Arg70(A), and Thr37(A). These interactions collectively confirm the specific and stable binding mode between the ligand and target protein (Figure 4d).

Figure 4Study on the mechanism of AA apply to human CSMC. (a) Research on molecular docking between AA and RhoA. The 3D structure of docking molecules bound to RhoA. (b) The amino acid residue LEU‐57 at the binding site of AA to RhoA. (c) Careful observation of the binding sites between AA and RhoA. (d) Other binding sites between AA and RhoA. (e) The RhoA/ROCK signaling pathway mediates AA‐induced cervical smooth muscle contraction by regulating MLC phosphorylation. Western blotting analysis of relative levels of MLC and p‐MLC in protein samples from AA treated or untreated human CSMC in the presence or absence of Y27632. (f) Densitometric analysis of western blots. (∗∗*p* < 0.01 and ∗∗∗*p* < 0.001).

**Figure:**
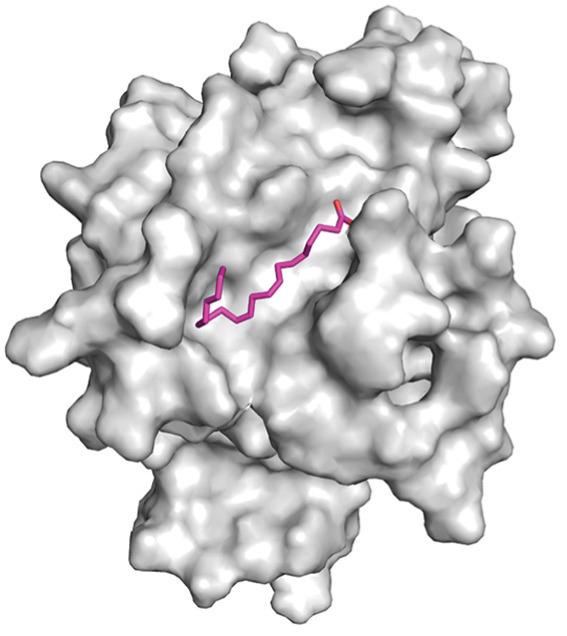
(a)

**Figure:**
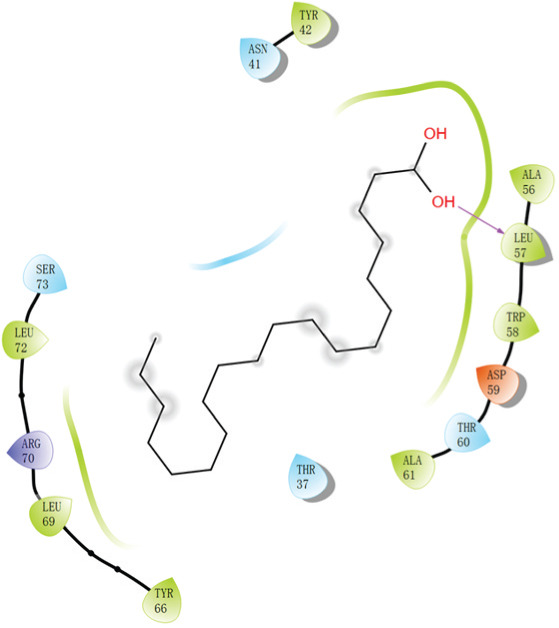
(b)

**Figure:**
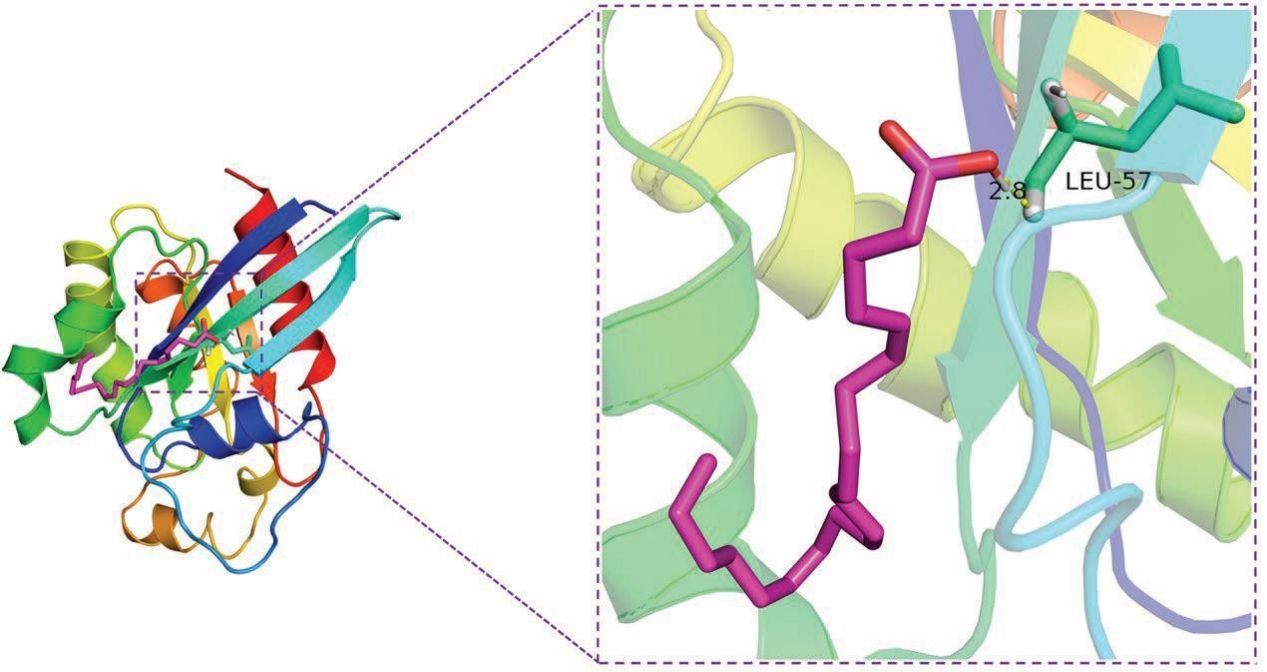
(c)

**Figure:**
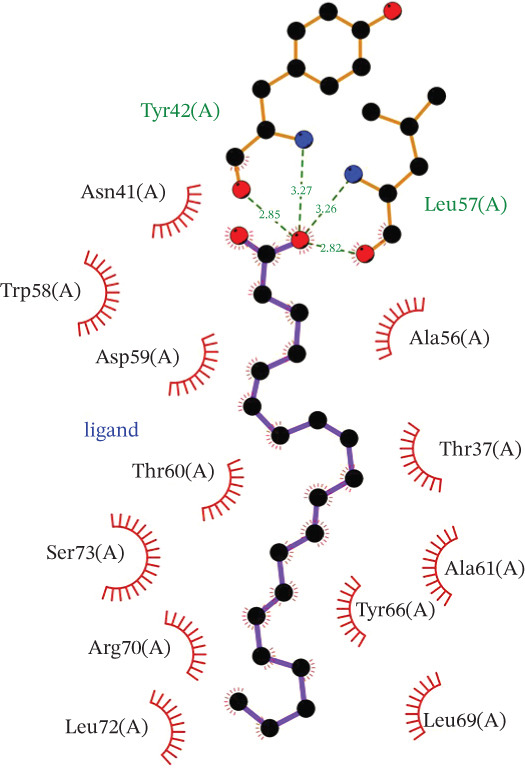
(d)

**Figure:**
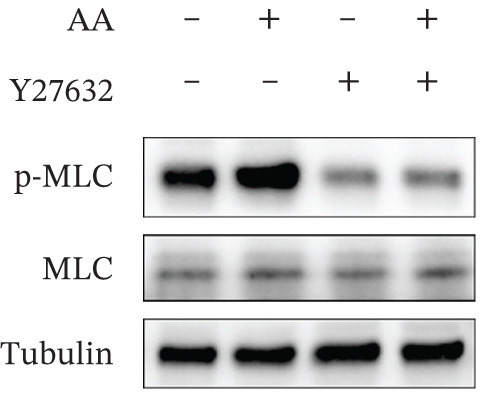
(e)

**Figure:**
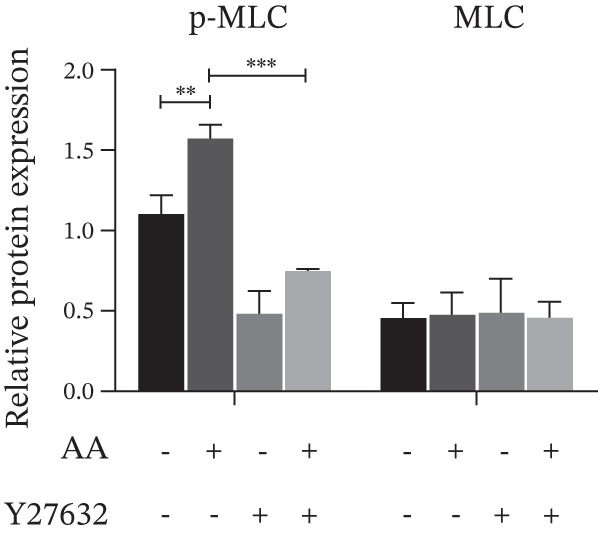
(f)

As shown in the results of Western Blot (Figure 4e), the RhoA/ROCK signaling pathway mediates AA‐induced cervical smooth muscle contraction by regulating MLC phosphorylation. It demonstrates the expression levels of MLC and p‐MLC from human CSMCs treated with or without AA, in the presence or absence of Y27632 (ROCK inhibitors), respectively. After treating CSMC with AA, the expression of p‐MLC was significantly increased, whereas the expression of p‐MLC decreased after Y27632 addition. The differences were statistically significant (Figure 4f). The result indicated that AA may play the biological role by promoting MLC phosphorylation.

## 4. Discussion

PTB defined as delivery at less than 37 weeks of gestation by the World Health Organization is estimated to affect about one in 10 pregnancies in the United States in 2021 [17]. The average incidence of PTB in China is 6.9%, and the number of infants born preterm reaches 1.17 million each year, ranking second to India among all countries [18]. The etiology of PTB has been explored for decades, such as very low or high body mass index of the mother, advanced age, smoking, previous history of PTB, cervical dysplasia, short cervical length, and infection. In addition, lower economic levels, literacy levels, and ethnic differences can also contribute to differences in adverse pregnancy outcomes [19], with inflammation considered as the most common mechanism. Increased inflammatory molecules such as IL‐1, TNF, and IL‐6 predicted the onset of PTB [20]. IL‐27 mediated the inflammatory cascade response through activation of the ERK signaling pathway, affecting the remodeling of fetal membranous tissue and contraction of the myometrium, and was involved in the onset of PTB [21]. In the cervical epithelium, hyaluronan (HA) is a key component of the mucosal barrier, regulating epithelial differentiation, cell connections, and resistance to ascending reproductive tract infections [22], whereas the matrix regulates tissue hardness through collagen remodeling and ECM recombination [23]. When epithelial barrier dysfunction (such as HA deficiency) allows microbial infiltration, leading to matrix inflammation and ECM breakdown, premature ripening arises. In mice, stimulation of TLR4 induced preterm labor, whereas TLR4 antagonists inhibited uterine contraction and reduced the production of proinflammatory cytokines [24]. Macrophages selectively accumulated before PTB [25], and the removal of macrophages may protect against endotoxin‐induced PTB [26]. Contraction of uterine smooth muscle is a sign of the onset of labor, and PTB resulting from a premature conversion of the myometrium (the thick smooth‐muscle layer of the uterus) from a quiescent to a contractile state [27], which tissue‐level inflammation is associated with myometrial muscle contraction [28].

Metabolites refer to small biological molecules that exist as precursors, intermediates, and final products throughout the entire cellular activity [29]. They can initiate cell signaling cascades, regulate various biological processes, and provide direct information on the state of the cell. Metabolites in the human body can be categorized into endogenous metabolites and exogenous metabolites. Endogenous metabolites refer to the small biological molecules that are produced by the life activities within the human body, whereas exogenous metabolites are those small biological molecules that are introduced into the human body through external means, such as diet. Typically, human endogenous metabolites encompass small molecule metabolites originating from cells, tissues, and body fluids. The study of human endogenous metabolites has been used for health assessment and disease diagnosis [30]. Biomarkers of PTB have been identified through metabolic profiles of amniotic fluid, maternal urine/maternal blood, and cervicovaginal fluid [31]. Multiple studies have found that four metabolites, 5‐oxoproline, creatinine, histidine, and myoinositol, are inversely correlated with PTB [32–34]. During pregnancy, the metabolites of vaginal discharge acetone, ethanol, ethylene glycol, formate, isopropanol, methanol, and trimethylamine N‐oxide (TMAO) increased significantly in the preterm group, which were beneficial markers for predicting PTB [35]. Plasma metabolic profiles verified the accuracy of 13‐cis‐retinoic acid (cis‐RA) in predicting PTB and played an important role in maintaining pregnancy and inducing PTB [36]. The expression level of the endogenous metabolite AA was significantly higher in the plasma of inflammatory mice, indicating AA may be involved in the occurrence of PTB. All these studies have shown that inflammation caused by metabolites is significantly associated with cascades that promote PTB.

In our previous research, we established a mouse model of PTB through LPS induction in the early stage, and the identification and study of mouse plasma metabolism profiles suggested that metabolites play an important role in maintaining pregnancy and inducing premature cervical maturation, and differentially expressed metabolites may cause premature cervical maturation, resulting in PTB [13]. We established the inflammation model of PTB mice, anatomically dissected the entire tissues, and found that compared with the normal group, the fetuses of the premature group were translucent and not fully developed. The immunohistochemical slides show that the cervical tissue of PTB mice contained a large amount of connexin‐43, OR, COX‐2, and calponin. Our findings align with prior research, providing robust evidence that the expression of contraction‐associated proteins is disrupted in PTB. This is consistent with known mechanisms where perturbations of connexin‐43, OR, and COX‐2 contribute to the early onset of CSMC contractions [13].

AA plays a key role in cardiovascular biology, carcinogenesis, and many inflammatory diseases, such as asthma and arthritis [37]. A wealth of evidence arising from mouse models as well as human studies supports that PTB results from excessive, premature inflammation reaction [38]. Although research has focused on the pathophysiology of PTB in recent years, the relationship between AA and CSMC, PTB and the specific mechanism of AA have not yet been explored. We detected that AA content in PTB group mice was higher than that in the control group, which was also consistent with the inflammatory phenotype change of PTB.

The RhoA/ROCK signaling pathway is downstream of AA and is reported to mediate metabolic‐cardio‐renal dysfunctions in some experimental models of insulin resistance and diabetes [39]. RhoA (a member of the Rho family of Ras homolog genes) and ROCK (Rho‐associated coiled‐coil kinase, known as Rho kinases, belong to the serine–threonine kinase family), mediate the induction of cytoskeletal reorganization, cell migration, adhesion, proliferation and differentiation, and tissue contraction. ROCK inhibitors have potential therapeutic applications in diseases such as asthma, cancer, erectile dysfunction, glaucoma, insulin resistance, renal failure, neurodegeneration, and osteoporosis [40]. After RhoA is activated, the ROCK protein acts as the main downstream effector, and the phosphorylation of MLC contributes to actin depolymerization and contractile activity, thereby regulating endothelial cell adhesion and the contraction of actomyosin [41]. Since thrombin stimulates phospho‐MLC through RhoA/Rho‐associated, ROCK‐dependent inhibition of MLC phosphates [42], we examined the effects of AA on this pathway. Our experimental data directly validate this regulatory axis: AA treatment of CSMCs significantly upregulates p‐MLC expression, whereas administration of the selective ROCK inhibitor Y27632 abrogates this effect. These findings confirm that the RhoA/ROCK pathway is a core mediator of AA‐induced CSMC contraction, representing the primary signaling cascade driving the contractile component of AA‐induced PTB.

Additionally, our model of PTB was induced by LPS. LPS is known to upregulate T‐type calcium channels in cervical tissues [43], which can potentiate intracellular calcium elevation—a classic trigger of smooth muscle contraction. This Ca^2+^/MLCK‐dependent mechanism may also contribute to cervical smooth muscle contraction. In this cascade, increased cytoplasmic Ca^2+^ binds to calmodulin, forming an active complex that activates MLCK, which directly phosphorylates MLC to initiate contractile responses. Importantly, this pathway does not antagonize the RhoA/ROCK axis but rather operates in a synergistic manner: the RhoA/ROCK pathway sustains MLC phosphorylation by inhibiting dephosphorylation, whereas the Ca^2+^/MLCK pathway provides a parallel, direct activation signal for MLC phosphorylation. However, the primacy of the RhoA/ROCK pathway is underscored by our data showing that targeted inhibition of ROCK is sufficient to abrogate AA‐induced p‐MLC upregulation, indicating that even in the presence of potential Ca^2+^‐dependent inputs, the RhoA/ROCK axis remains the dominant regulator of contractile signaling in this context.

Prior studies have demonstrated that AA directly upregulates matrix metalloproteinase 9 (MMP‐9) expression [44], a key matrix metalloproteinase that degrades ECM components (e.g., collagen and laminin) to facilitate cervical remodeling and softening. Additionally, elevated connexin‐43 expression has been linked to increased MMP‐9 production [45], suggesting a connexin‐43–MMP‐9 axis that contributes to cervical ripening. Based on the above research and our experimental results, we speculate that MMP‐9–induced degradation of the cervical ECM reduces tissue mechanical resistance, enabling the contractile force generated by the RhoA/ROCK pathway to more effectively induce cervical dilatation and preterm delivery. However, the RhoA/ROCK pathway remains the central functional effector, as cervical ripening alone—without active smooth muscle contraction—cannot drive the cervical dilatation of PTB.

**Figure 5:**
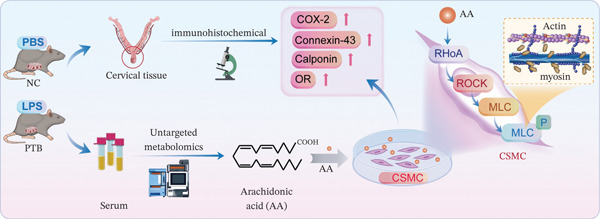
Flow chart of the research. The figure illustrates the experimental protocol. Pregnant C57BL/6 mice were treated with LPS or PBS. Blood serum and cervical tissue samples were taken from both PBS mice and LPS mice. Then, untargeted metabolomics analysis, immunohistochemical staining, western blot, and qPCR test were performed. After screening the differentially expressed metabolites, human CSMC were used to explore the potential functions and mechanism of AA.

In conclusion, our findings indicated that the metabolic profile of PTB mice was altered, with AA showing significantly higher expression in the PTB group. AA may promote cervical smooth muscle contraction, thereby contributing to PTB, possibly by inducing MLC phosphorylation via the RhoA/ROCK signaling pathway (Figure 5). Collectively, this study provides new insights into the pathogenesis of PTB, suggesting that CSMC contraction may be involved in PTB occurrence. Furthermore, the differentially expressed bioactive metabolite AA might represent a potential candidate therapeutic target for the prevention or treatment of PTB.

## Author Contributions

Xinyi Chen and Jing Chen have contributed equally to this work.

## Funding

This study was supported by the Youth Project of Changning District Health Commission (2022QN05) and Key Discipline Project of Shanghai Municipal Health System (2024ZDXK0064).

## Conflicts of Interest

The authors declare no conflicts of interest.

## Data Availability

The data that support the findings of this study are available on request from the corresponding authors. The data are not publicly available due to privacy or ethical restrictions.
